# A study on the morphologic change of palatal alveolar bone shape after intrusion and retraction of maxillary incisors

**DOI:** 10.1038/s41598-020-71115-6

**Published:** 2020-09-02

**Authors:** Eun Jeong Son, Soo Jin Kim, Christine Hong, Vania Chan, Hye Young Sim, Suk Ji, Seok Yoon Hong, Un-Bong Baik, Jeong Won Shin, Young Ho Kim, Hwa Sung Chae

**Affiliations:** 1 Department of Orthodontics, Institute of Oral Health Science, Ajou University School of Medicine, Suwon, South Korea; 2 Division of Orthodontics. School of Dentistry, University of California, San Francisco, CA USA; 3 School of Dentistry, University of California, Los Angeles, CA USA; 4 Department of Dentistry, SMG-SNU Boramae Medical Center, Seoul, South Korea; 5 Department of Periodontology, Institute of Oral Health Science, Ajou University School of Medicine, Suwon, South Korea

**Keywords:** Biological techniques, Biophysics, Biotechnology

## Abstract

The purpose of this study is to evaluate the changes in the palatal alveolar bone thickness and find the factors related to the resorption of the palatal alveolar bone caused by tooth movement after the maxillary incisors were retracted and intruded during orthodontic treatment. The study group comprised of 33 skeletal Class II malocclusion patients who underwent extraction for orthodontic treatment. Palatal alveolar bone thickness changes and resorption factors were identified and analyzed. The changes of maxillary central incisors and palatal alveolar bone thickness were measured, and the corresponding sample *t* test was performed using SPSS (IBM SPSS version 22). The amount of palatal alveolar bone resorption was measured and various parameters were analyzed to determine which factors affected it. Correlation analysis adopting the amount of palatal alveolar bone resorption as a dependent variable demonstrated that the SNB, mandibular plane angle, and the inclination of the maxillary central incisor were significantly correlated with before treatment. On the other hand, mandibular plane angle, angle of convexity, the inclination of the upper incisor, and the occlusal plane (UOP, POP) were significantly correlated with post-treatment. In addition, the variables related to palatal contour (PP to PAS, SN to PAS, palatal surface angle) and occlusal planes (UOP/POP) were significantly correlated with the difference in palatal bone resorption. During initial diagnosis, high angle class II with normal upper incisor inclination can be signs of high-risk factors. In addition, maintaining the occlusal plane during treatment helps to prevent palatal bone loss.

## Introduction

Patients seeking orthodontic treatment to resolve bimaxillary protrusion usually desire to change facial features which is important in establishing confidence and improving quality of life. To resolve these chief complaints, extraction of 4 premolars is usually required and surgery may also be utilized in cases when orthodontics only treatment is inadequate. Recent universalization of the temporary anchorage devices (TADs) and mini-plates has led to more stable and esthetic facial improvements through skeletal anchorage in orthodontic treatment^1–3^.

However, if there is excessive lingual inclination of anterior teeth as a result of putting too much emphasis on the esthetic aspect without considering the alveolar bone remodeling response following orthodontic tooth movement, unwanted iatrogenic sequelae such as root resorption, alveolar bone loss and fenestration, dehiscence and gingival recession would likely occur^4–10^.

Orthodontic tooth movement is a process where application of a force induces bone resorption on the pressure side and bone apposition on the tension side. Alexder et al. mentioned that a basic axiom in orthodontics is “Bone traces tooth movement,” suggesting that whenever orthodontic movement occurs, the bone around the alveolar socket will remodel to the same extent^11^. However, there is evidence that this premise does not hold true in the anterior region^11^. The excessive force causing fenestration and dehiscence might lead to alveolar bone loss, and there is disagreement about whether the remodeling capacity of the alveolar bone can compensate for the bone loss in every case^1^.

Among various studies investigating root resorption and alveolar bone loss, the study on the factors affecting root resorption of maxillary central incisors showed that the extraction group showed more root resorption compared to the non-extraction group and the difference was statistically significant^12^.

Other research investigating the relationship between the amount of tooth movement and the amount of bone remodeling during retraction of upper incisors after the extraction both in the tip-group and the torque-group showed that the amounts were not the same. The authors also suggested that there were limitations in the amount of incisor retraction in patients with a very thin anterior cortical plate in the maxilla, thus in patients with severe skeletal discrepancies, orthognathic surgery should be considered^13^. Also, the studies on the labial and palatal alveolar bone thickness of incisors after incisor retraction showed more significant changes in palatal bone compared to the labial side^4,5,10^.

Concerning palatal bone repair, Ahn^5^ noticed no repair at the debonding stage. On the other hand, Bae^1^ reported that a skeletal class II case patient who underwent maxillary incisor intrusion and retraction extraction treatment presented with palatal bone dehiscence at the end of treatment. However, 10 years later, the follow-up CT revealed palatal bone apposition. In this study, CT scans of the patients with longer than 5 years of retention were evaluated to investigate palatal bone status.

In summary, previous studies have shown that extraction treatment caused more root resorption and alveolar bone level change compared to non-extraction treatment, and also the alveolar bone thickness decreased significantly more on the palatal side compared to the labial side^4,5,14,15^.

There are few studies investigating the factors affecting the significant resorption of the palatal alveolar bone nor the relationship between palatal alveolar bone thickness and retraction of teeth.

The purpose of this study is to evaluate the changes in the palatal alveolar bone thickness and to determine the factors related to the resorption of the palatal alveolar bone caused by tooth movement when the maxillary incisors were retracted and intruded during orthodontic treatment.

## Materials and methods

### Study sample

The present study was approved by the institutional review board (IRB) of the Ajou University Hospital (IRB No: AJIRB-MED-MDB-18-295), and consent forms were filled out. 33 Korean female patients (mean age 25.8 years, from 14 to 49 years) who underwent extraction orthodontic treatment accompanied with upper incisor intrusion and retraction were examined. Inclusion criteria were (1) skeletal Class II with maxillary incisor protrusion (3° < ANB < 11.5°, U1toN-A > 4 mm), (2) overjet/overbite (2 < OJ < 5 mm, 2 < OB < 3 mm), (3) mild crowding (arch length discrepancy less than 3 mm), (4) four first premolars were extracted, and (5) TADs were used to retract and intrude upper incisors. Patients with medical history related to bone metabolism, with history of taking anti-inflammatory drugs during treatment or within 6 months before treatment, with periodontal or gingival diseases at the beginning of orthodontic treatment, and with a trauma history of maxillary incisors were excluded.

All the patients were treated by one clinician. 0.022″ MBT brackets were bonded, followed by sequential wire changes adopting 0.016″ Nickel titanium, 0.018 × 0.025″ Bioforce (Densply Sirona, USA), and 0.019 × 0.025″ Stainless-Steel for the working wire. Both sliding and loop mechanics were used. For most of the cases, the maxillary 1st premolars were extracted, and the maximum anchorage was prepared. The treatment duration of incisor retraction was 6–9 months. For intrusion and retraction of the maxillary incisors, since the center of resistance of the maxillary anterior six teeth is closely located between upper lateral incisors and canines^16,17^, TADs (temporary anchorage devices) were inserted there in most cases unless those TADs failed.

### Cephalometric measurement

Lateral cephalograms were taken at the natural head position, and all measurements were analyzed by one researcher using V-ceph 7.0 digital program (Cybermed, Seoul, South Korea). To verify measurement error, repeated tracings and measurements were performed at a 2-week interval on ten randomly selected patients. Measurement error was estimated for two sets of data using Dahlberg’s formula^18^. The differences were statistically insignificant.

**Figure 1:**
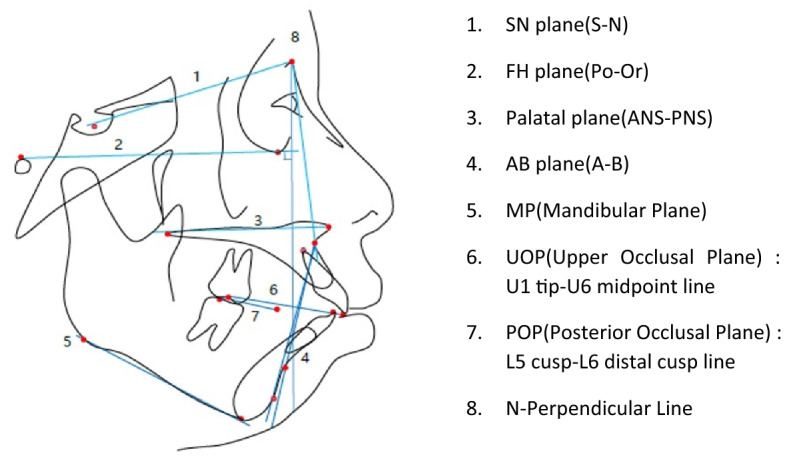
Landmarks and reference lines used for the cephalometric analysis.

1. The pretreatment and posttreatment lateral cephalometric radiographs were taken, and a total of 8 reference planes, 16 linear and angular variables which could affect the palatal alveolar bone remodeling of upper incisors were measured (Fig. 1, Table 1).

**Table 1:** Linear and angular variables which could affect the palatal alveolar bone remodeling of upper incisors.

Skeletal measurement	Explanation
Angular : SNA	Angle of nasion-sella to nasion-A point
SNB	Angle of nasion-sella to nasion-B point
ANB	Angle of A point-nasion to nasion-B point
Sum	Sum of saddle angle + articular angle + gonial angle
Linear: SE-PNS	Posterior maxillar height
N-ANS	Anterior maxillar height
A point-N	Perpendicular maxillar anterior–posterior relative to N

Dental measurement	Explanation
Angular : FH-U1	Angle of FH to U1 axis
SN-U1	Angle of SN to U1 axis
IIA	Angle of inter-incisal
Y-axis	Angle of FH to sella-constructed gnathion
AB plane angle	Angle of FH to A-B plane
Angle of convexity	Angle of A-nasion to nasion-pogonion
MP	Angle of FH to mandibular plane
IMPA	Angle of MP to L1 axis
Wits	Distances between the points of contact of the perpendicular lines on the occlusal plane(AO;A point the OP/BO;B point the OP)
PP-U1	Angle of palatal plane to U1 axis
PP-PAS	Angle of palatal plane to palatal alveolar surface line
PAS-U1 axis	Angle of palatal alveolar surface to U1 axis
Palatal surface angle	Angle of palatal horizontal and vertical surface line
UOP (upper occlusal plane)	Angle of FH to U1 tip-u6 midpoint line
POP (posterior occlusal plane)	Angle to FH to L5 cusp tip-L6 distal cusp line
2. The amount of retraction and intrusion of maxillary incisors were included (Fig. 2).

**Figure 2:**
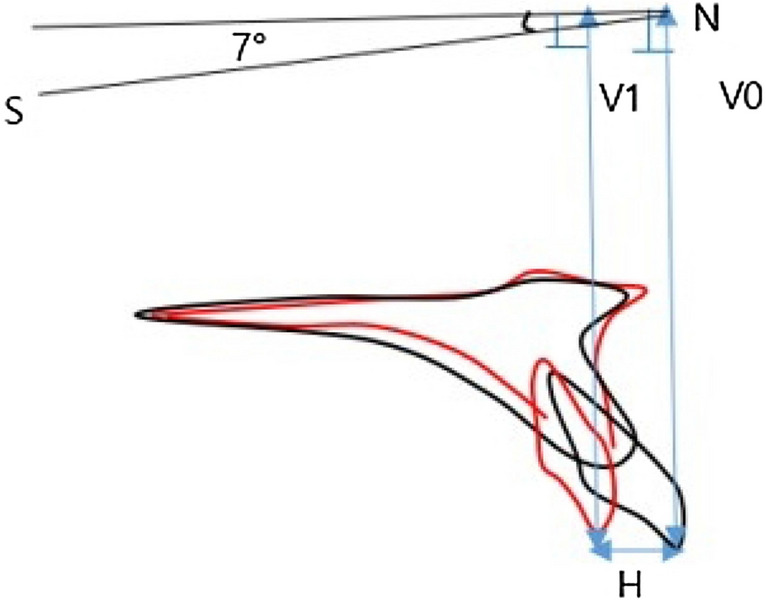
The amount of intrusion (difference between V0 and V1), and retraction (H) of maxillary incisors.
3. To evaluate alveolar bone remodeling on the labial side and palatal side, the angle between U1-LASL (labial surface line of maxillary central incisor) and LAS (labial alveolar bone surface) before and after the treatment (Fig. 3-1a in in Fig. 3) and the angle between U1-LISL (lingual surface line of maxillary central incisor) and PAS (palatal alveolar bone surface) before and after the treatment (Fig. 3-1b in Fig. 3) were measured respectively. The most tangent line was drawn with the following sequence. First, FH to Nasion perpendicular line was established as a vertical reference line. The closest point from the line to the alveolar bone or the maxillary central incisor was set as the original point. Next, a protractor was used to find the tangent point, which is the point where the protractor first meets the superior structure from the origin. The tangent line was then drawn from those two points. Figure 3-1 in Fig. 3 demonstrates how the angle between the two tangent lines are measured. To evaluate palatal bone contour and its relationship to palatal plane and the maxillary central incisor, variables are named and measured (Fig. 3-b in Fig. 3, Table 1).

**Figure 3:**
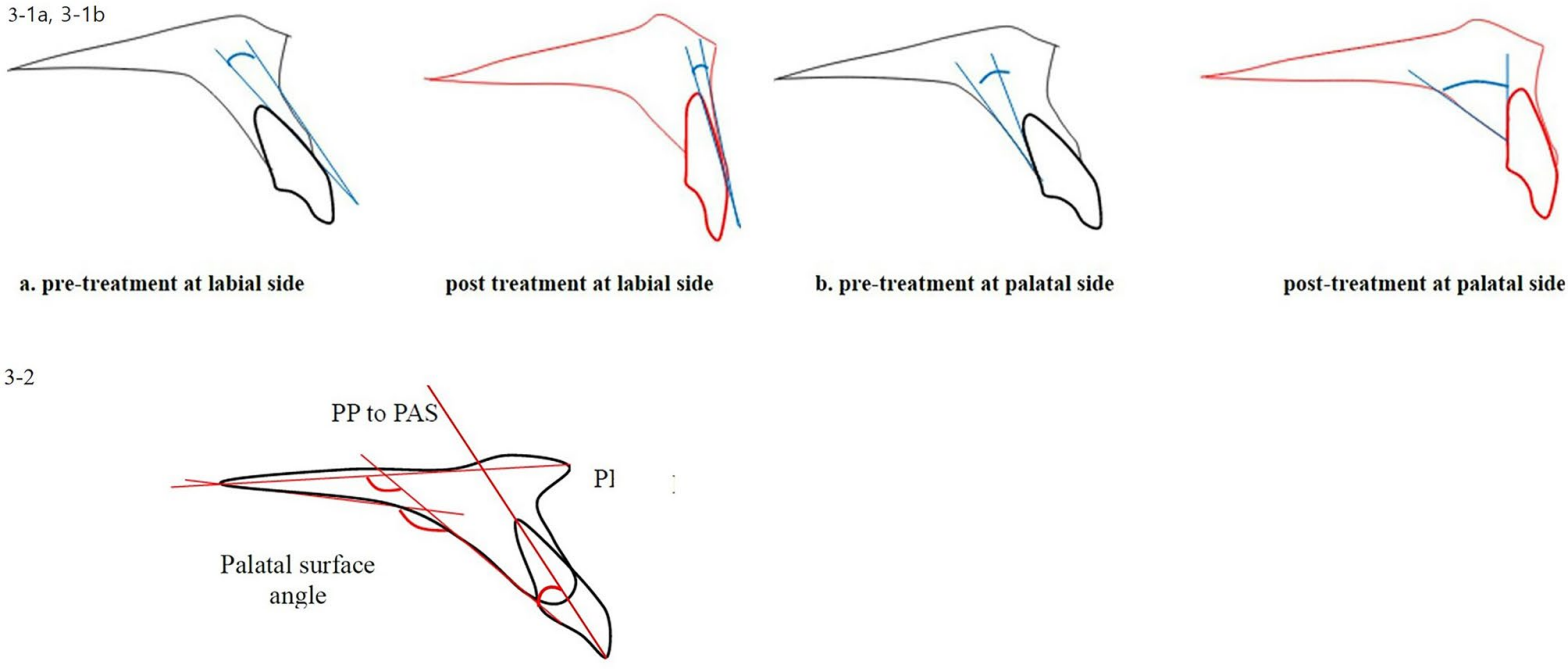
(**3-1**) The angle between U1 (maxillary central incisor) and alveolar bone surface before and after the treatment; (**a**) the angle between U1-LAS (labial surface line of maxillary central incisor) and LAS (labial alveolar bone surface) before and after treatment. (**b**) The angle between U1-L1 (lingual surface line of maxillary central incisor) and PAS (palatal alveolar bone surface) before and after treatment. (**3-2**) Palatal contour measurements; *PP-PAS* angle of palatal plane to palatal alveolar surface line; *PAS-U1 axis* angle of palatal alveolar surface to U1 axis; *Palatal surface angle* angle of palatal horizontal and vertical surface line.
4. To evaluate the amount of maxillary incisor retraction and the change of palatal bone thickness, horizontal reference plane (HRP), as was used in the previous studies^5,15^, a plane angulated 7° upward to the SN plane passing through Nasion, and vertical reference plane (VRP), a perpendicular plane to the HRP passing through Nasion were used.
  The amount of retraction of the maxillary central incisor was measured as the distance from VRP at 0, 2 mm, 4 mm, 6 mm, and 8 mm upward from the CEJ (Cemento-Enamel-Junction). The corresponding measured amount was indexed as R1, R2, R3, R4, and R5, respectively, with R1 being at the CEJ (Fig. 4).

**Figure 4:**
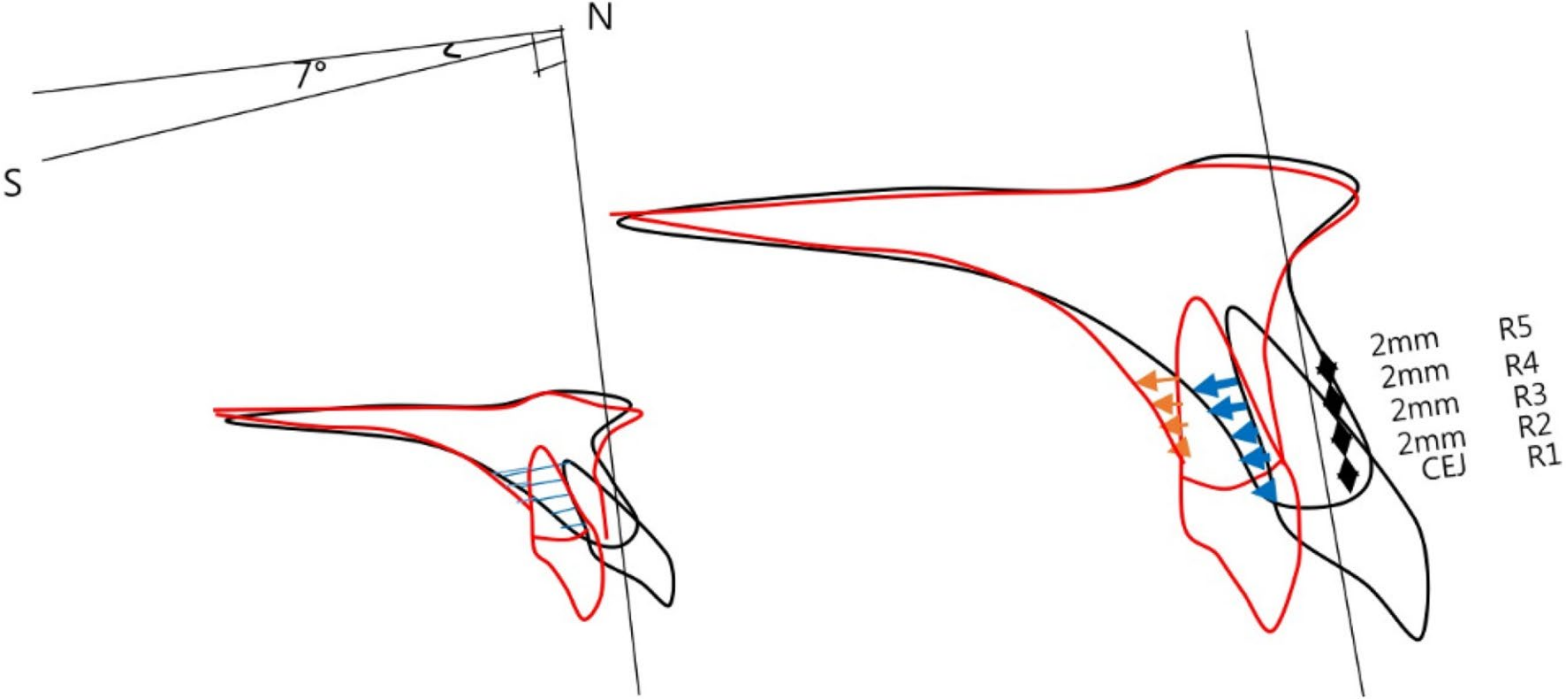
The amount of retraction measurements.
  Likewise, the thickness of palatal alveolar bone before and after the treatment was measured at the same points and indexed as T1, T2, T3, T4, and T5 respectively, with T1 being at the CEJ (Fig. 5).

**Figure 5:**
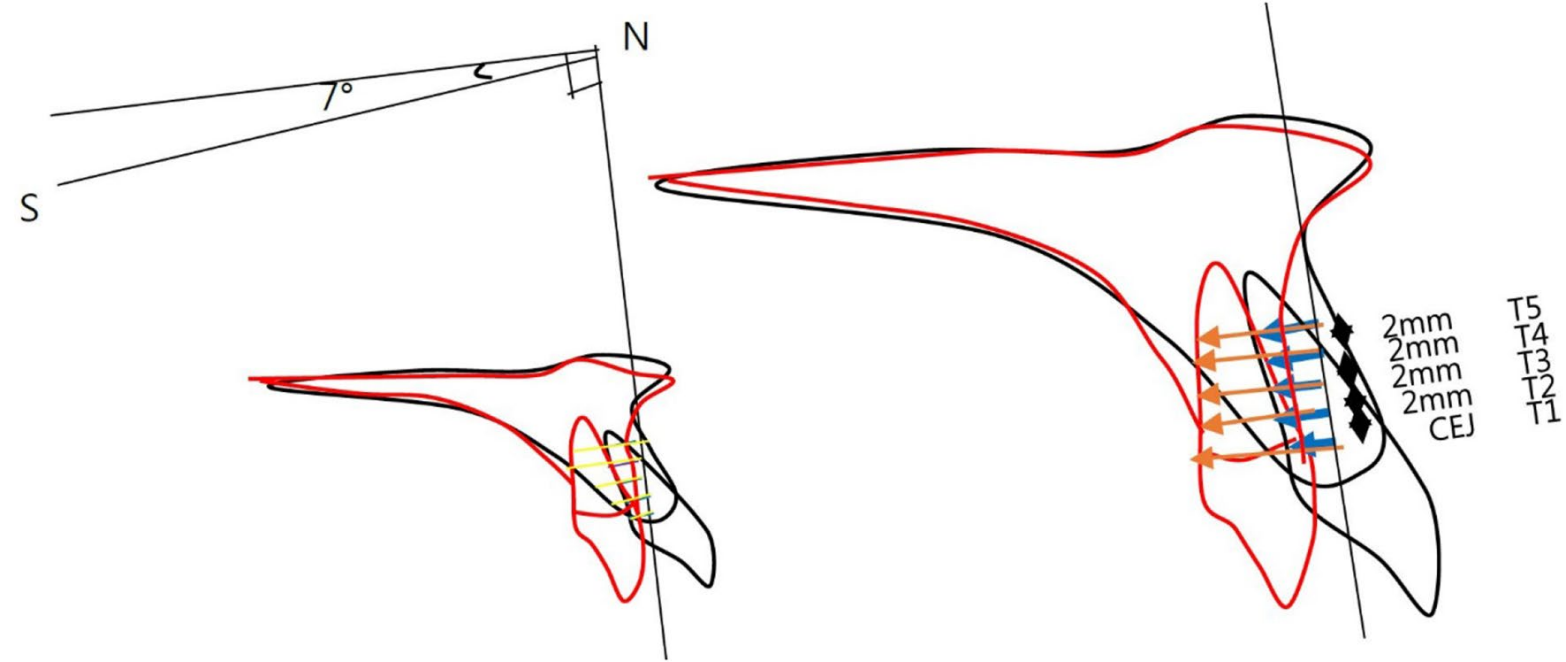
The thickness of palatal alveolar bone measurements.
5. The amount of vertical resorption of palatal alveolar bone was measured as the difference between the distances from HRP to the marginal point of palatal bone before and after the treatment (Fig. 6).

**Figure 6:**
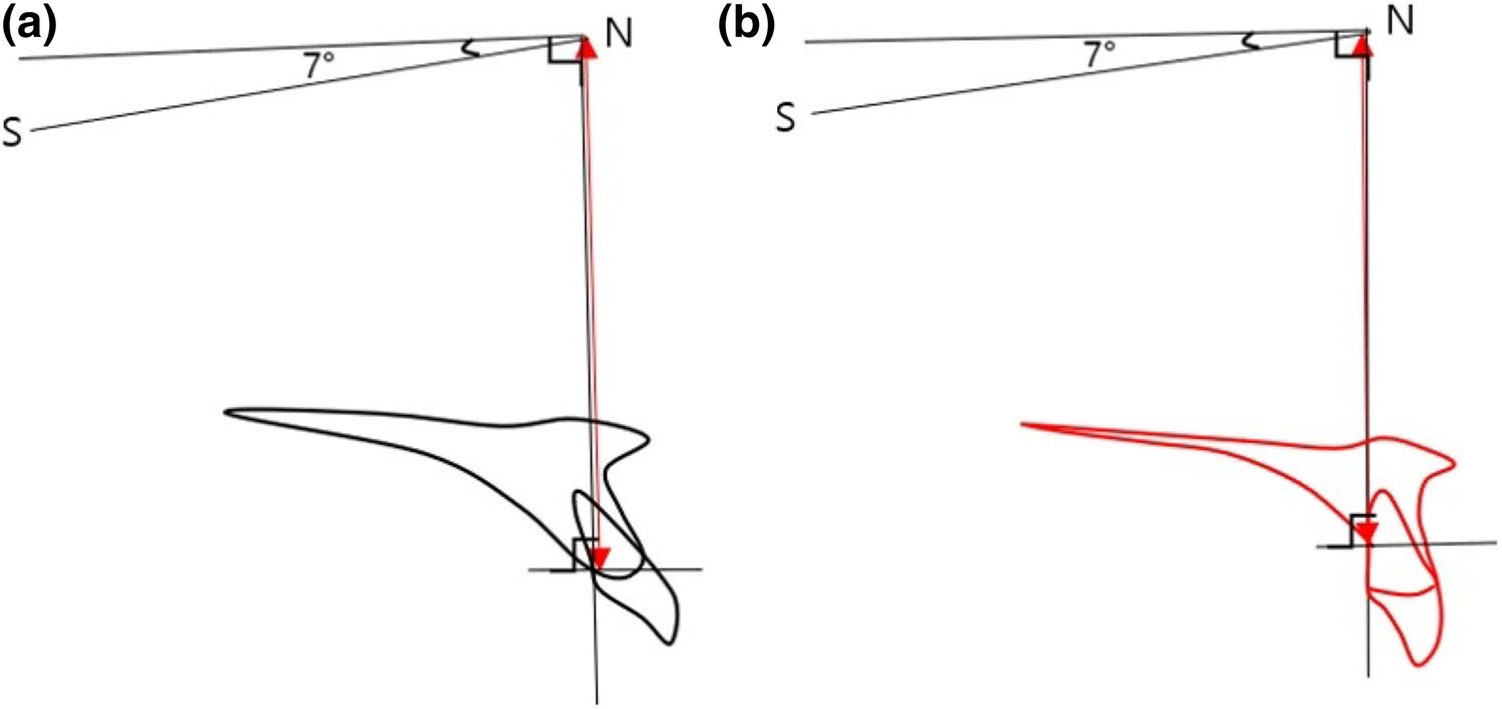
The amount of palatal alveolar bone resorption at (**a**) pre-treatment and (**b**) post-treatment.
6. To measure the palatal alveolar bone resorption, the surface area of palatal alveolar bone was measured using the points which make up the distances from incisor tip to root tip the same (a = a′) before and after the treatment (Fig. 7).

**Figure 7:**
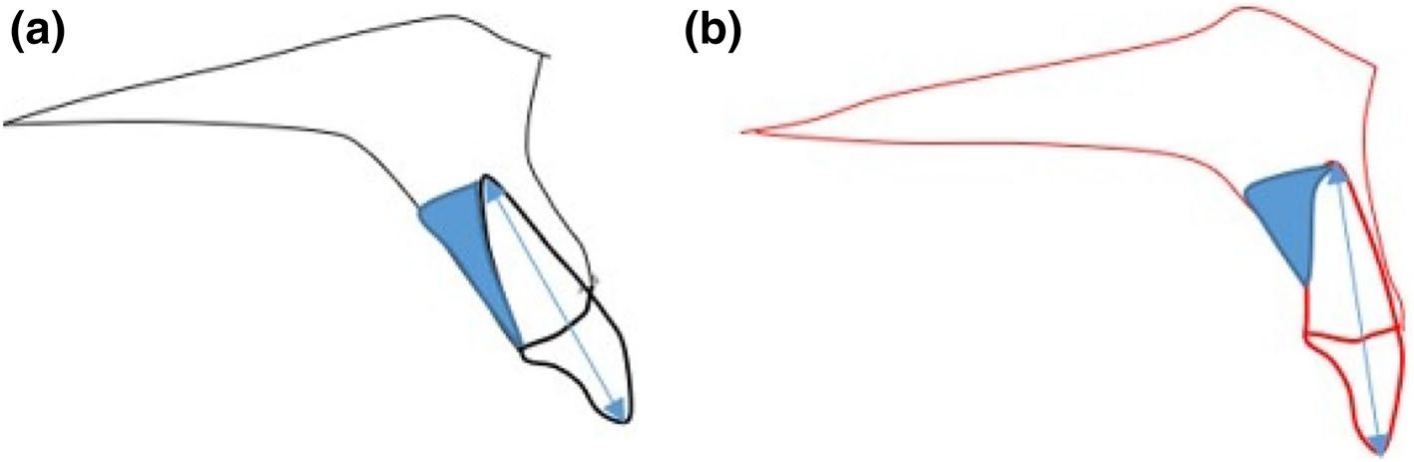
The surface area of palatal alveolar bone (**a**) before and (**b**) after the treatment.
7. The angular and linear variables expected to affect alveolar bone remodeling following maxillary incisor retraction were selected, and the differences between pre-treatment and post-treatment (DF-treatment) were calculated (Table 1).

All the distances were measured as pictures magnified three times for the accuracy, followed by shrunken back to the original 1:1 scale. All measurements were performed twice by a single investigator at a 4-week interval.

Maspero^19^ suggested that CBCT is essential to investigate palatal bone change after orthodontic treatment. Vasconcelos^20^ indicated that CBCT is the only way to detect the buccal and palatal surface of alveolar bone. The detection of bony defects using CBCT was as accurate as in vivo measurements performed during the periodontal surgical treatment^21^. One of the aims of the present study was to assess whether repair of the palatal alveolar bone during the retention period occurs or not. Thus, the examination of each anterior tooth was conducted through CBCT. Among 33 patients, 11 patients with retention longer than 5 years were asked to return to take a conebeam CT scan (Pax3iD, Vatech, Korea). The deepest point from the CEJ to alveolar bone margin was measured following a previous study^20^ with EZ3iD software (Vatech, Korea).

### Statistical analysis

Statistical analyses were performed with the SPSS program (IBM SPSS version 22) for the paired *t* test and Pearson correlation analysis.

1. Paired *t* test at a 0.05 significance level was used to evaluate the movement of maxillary central incisors and the changes of the angle between the palatal alveolar bone and maxillary central incisor, as factor index of the alveolar bone remodeling after retraction and intrusion of incisors.
2. The amount of retraction of maxillary incisors and the change in palatal alveolar bone thickness at R1–R5 were tested using the paired *t* test, and the ratio of change was investigated.
3. Pearson correlation analysis was applied for palatal alveolar bone thickness, from T1 to T5.
4. Pearson correlation coefficients were measured to identify variables related to alveolar bone remodeling with a significance level of 0.05, setting palatal alveolar bone resorption as the dependent variable and change in angle before and after the treatment, surface area, intrusion, and age as independent variables.

### Ethics approval and consent to participate

This study was approved by the institutional review board (IRB) of the Ajou University Hospital (IRB No: AJIRB-MED-MDB-18-295). Subjects read and signed their informed consents. The authors are confirming all the experiment protocol for involving humans was in accordance to guidelines of national/international/institutional or Declaration of Helsinki.

## Results

### Comparison of alveolar bone remodeling in the labial side and palatal side (Table 2, Fig. 3)

The alveolar bone on the labial side of the maxillary central incisor showed less angular change relative to the surface of upper incisors, which indicates more remodeling compared to the alveolar bone on the palatal side.

**Table 2:** The comparison of alveolar bone remodeling at labial side and palatal side.

Paired sample								
Paired difference								
95% confidence level of difference								
	Mean	SD	SD mean	Lower end	Upper end	t	df	p value(both sides)
Pair labial palatal	8.546	5.799	1.01	10.602	6.489	8.465	32	0.00

Paired *t* test								
	Pretreatment (mean ± SD)	Posttreatment (mean ± SD)	Difference (mean ± SD)	p value				
**Pair**								
Labial	6.5 ± 2.8	6.7 ± 2.0	2.2 ± 1.6	0.000*				
Palatal	24.0 ± 4.5	34.5 ± 5.8	10.7 ± 6.0	0.000*				

### The amount of maxillary central incisor retraction and the changes of palatal alveolar bone thickness during treatment (Tables 3, 4)

The change in the movement of the area up to 8 mm from the CEJ of the maxillary central incisor before and after treatment showed an average controlled tipping.

**Table 3:** Paired *t* test of maxillary central incisor retraction.

R	Pretreatment		Posttreatment		p value
	Mean	SD	Mean	SD	
R1	3.700	2.352	6.463	2.412	0.000***
R2	4.142	2.358	6.496	2.401	0.000***
R3	4.473	2.315	6.555	2.375	0.000***
R4	4.758	2.282	6.538	2.341	0.000***
R5	4.871	2.328	6.471	2.363	0.000***

*p < 0.05, **p < 0.01, ***p < 0.001.

**Table 4:** Paired *t* test of palatal alveolar bone thickness.

T	Pretreatment		Posttreatment		p value
	Mean	SD	Mean	SD	
T1	1.268	0.543	0.247	0.465	0.000***
T2	2.081	0.752	0.799	0.737	0.000***
T3	2.920	0.989	1.757	1.092	0.000***
T4	3.881	1.216	2.996	1.264	0.000***
T5	4.793	1.413	4.400	1.432	0.051

*p < 0.05, **p < 0.01, ***p < 0.001.

The change of the palatal alveolar bone thickness following the retraction of maxillary central incisors was measured, and the correspondence sample *t* test analysis showed that R1–R5 moved 2.8 mm, 2.4 mm, 2.1 mm, 1.7 mm, 1.6 mm, on average, and T1–T5 reduced thickness by 0.8 mm, 0.61 mm, 0.39 mm, 022 mm, and 0.08 mm, respectively, based on the thickness of the calibration before and after the treatment and showed an average controlled tipping.

The change of palatal alveolar bone thickness showed that alveolar bone was absorbed more toward the cervical region.

### The rate of change of palatal alveolar bone thickness following the retraction of maxillary incisors (Table 5)

At the CEJ, R1, the alveolar bone thickness decreased 0.805 mm with 2.76 mm retraction, representing a ratio of change of − 29.1%. At R2, the ratio of change was − 26.1%, at R3 was − 19.1%, at R4 was − 12.8%, and R5 was − 12.8% respectively. From these results, it can be conjectured that the alveolar bone resorption was more profound around the CEJ irrespective of retraction amount.

**Table 5:** The ratio of change in thickness compared to maxillary central incisors.

R	Mean difference	Thickness	ΔT/T mean	Ratio (%)
R1	2.762	T1	− 0.805	− 29.1
R2	2.354	T2	− 0.615	− 26.1
R3	2.082	T3	− 0.398	− 19.1
R4	1.781	T4	− 0.228	− 12.8
R5	1.6	T5	− 0.082	− 5.1

### Correlation analysis for palatal alveolar bone thickness from T1 to T5 (Table 6)

Based on the significant difference of the ratio of change from T1–T4, we can infer that thinner palatal bone before treatment, in general, results in more palatal alveolar bone resorption. Furthermore, correlation between the levels of thickness revealed that consistent thickness change can be expected on the palatal side.

**Table 6:** Correlation analysis of palatal alveolar bone thickness from T1 to T5.

	ΔT1	ΔT2	ΔT3	ΔT4	ΔT5
Pretreatment T1	− 0.694*** (0.000)	− 0.403* (0.002)	− 0.343 (0.051)	− 0.217 (0.225)	− 0.015 (0.935)
Pretreatment T2	− 0.741*** (0.000)	− 0.598*** (0.000)	− 0.402* (0.019)	− 0.295 (0.096)	0.036 (0.843)
Pretreatment T3	− 0.597*** (0.000)	− 0.417* (0.016)	− 0.382* (0.028)	− 0.32 (0.070)	− 0.138 (0.444)
Pretreatment T4	− 0.617*** (0.000)	− 0.434* (0.012)	− 0.363* (0.038)	− 0.381* (0.029)	− 0.183 (0.308)
Pretreatment T5	− 0.457*** (0.000)	− 0.181 (0.313)	− 0.168 (0.359)	− 0.203 (0.256)	− 0.376* (0.031)
ΔT1	1	0.724*** (0.000)	0.554** (0.001)	0.409* (0.018)	0.137 (0.449)
ΔT2	0.724*** (0.000)	1	0.817*** (0.000)	0.684*** (0.000)	0.164 (0.363)
ΔT3	0.554** (0.001)	0.817*** (0.000)	1	0.872*** (0.000)	0.501** (0.003)
ΔT4	0.409* (0.018)	0.684*** (0.000)	0.872*** (0.000)	1	0.658*** (0.000)
ΔT5	0.137 (0.449)	0.164 (0.363)	0.501** (0.003)	0.658*** (0.000)	1

*p < 0.05, **p < 0.01, ***p < 0.001.

### Factors affecting the vertical resorption of alveolar bone (Table 7)

According to the correlation analysis result, when the amount of vertical palatal alveolar bone resorption is analyzed (Fig. 6) as a dependent variable, SNB, mandibular plane angle, and inclination of maxillary incisors were significantly correlated with pre-treatment. On the other hand, mandibular plane angle, angle of convexity, the inclination of the maxillary incisors, occlusal plane (UOP, POP), and posterior palatal surface area (Fig. 7) were significantly correlated with post-treatment. Furthermore, the variables related to palatal contour (PP to PAS, SN to PAS, palatal surface angle) and occlusal planes (UOP/POP) were significantly correlated with the difference of palatal bone resorption between pre and post-treatment (Table 7).

**Table 7:** Correlation analysis between vertical palatal alveolar bone resorption and pre/post/difference values.

Initial (pre) values	Pearson coefficient p value (both sides)	Final (post) values	Pearson coefficient p value (both sides)	Difference (pre − post)	Pearson coefficient p value (both sides)
Pre SNB	0.451*	Post SNB	− 0.462**	DIF UOP	0.522**
	0.008		0.007		0.002
Pre MP	0.362*	Post MP	0.404*	DIF POP	0.617***
	0.038		0.020		0.000
Pre PAS to U1 axis	0.545**	Post PAS to U1 axis	0.635***	DIF PP to PAS	0.676***
	0.001		0.000		0.000
Pre surface area	− 0.344*	Post surface area	0.405*	DIF SN to PAS	0.486**
	0.050		0.019		0.004
Pre U1 to SN	− 0.451*	Post U1 to SN	− 0.480**	DIF palatal surface angle	0.583***
	0.008		0.005		0.000
Age	0.005	Post UOP	0.479**	DIF intrusion	− 0.121
	0.997		0.005		0.526
		Post POP	0.563**	DIF retraction	0.001
			0.001		0.998
		Post PP to PAS	0.512**		
			0.002		
		Post palatal surface angle	0.524**		
			0.002		
		Post angle of convexity	0.430*		
			0.013		

*p < 0.05, **p < 0.01, ***p < 0.001.

### Follow up of patients with retention longer than 5 years

**Figure 8:**
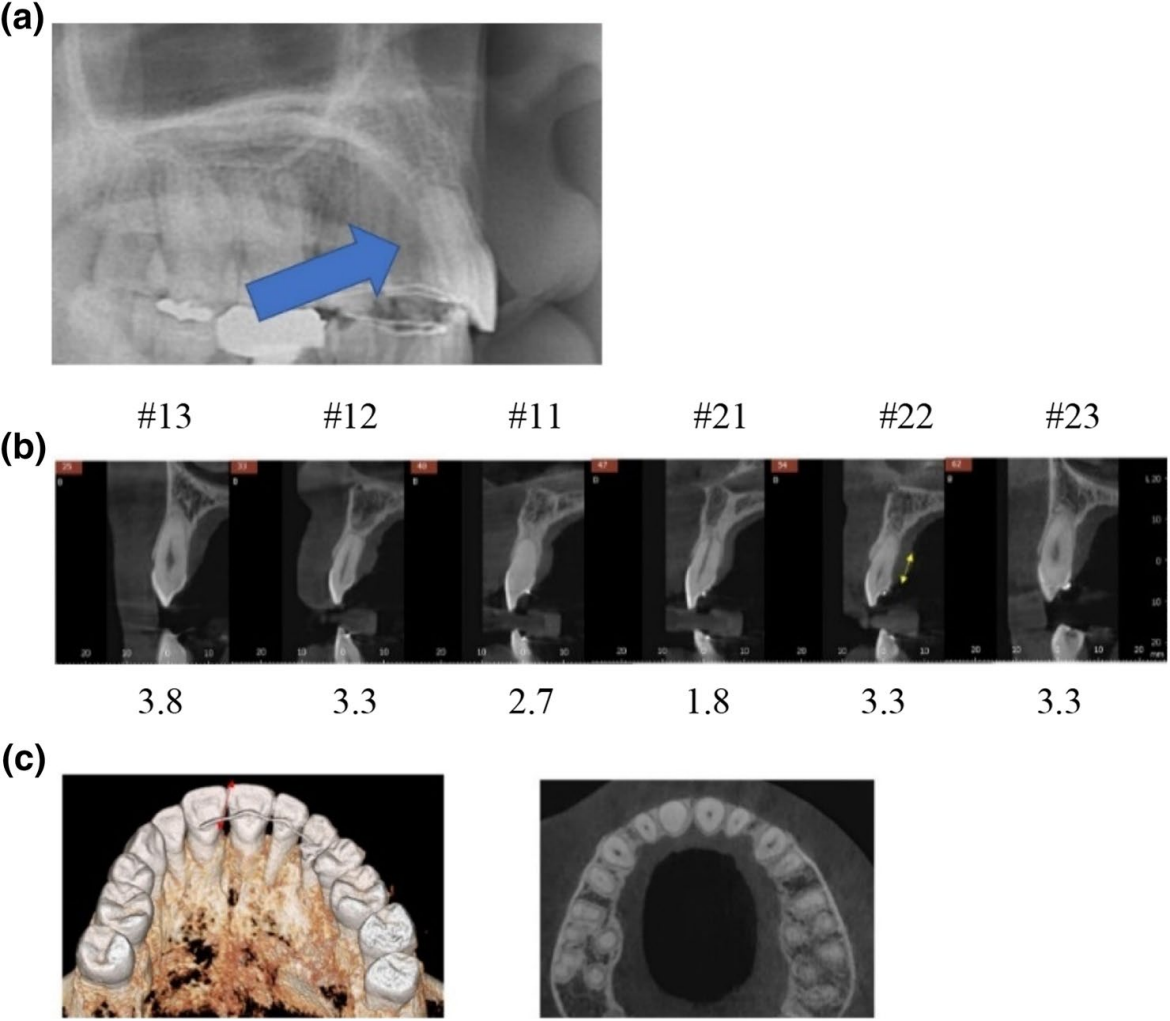
A case longer than 5 years of retention; (**a**) Debonding stage. Arrow indicates palatal bone loss; (**b**) numbers below the teeth indicate the distance from CEJ to the deepest point of alveolar crest; (**c**) the transverse view of the alveolar bone thickness after 5 years of retention.

**Figure 9:**
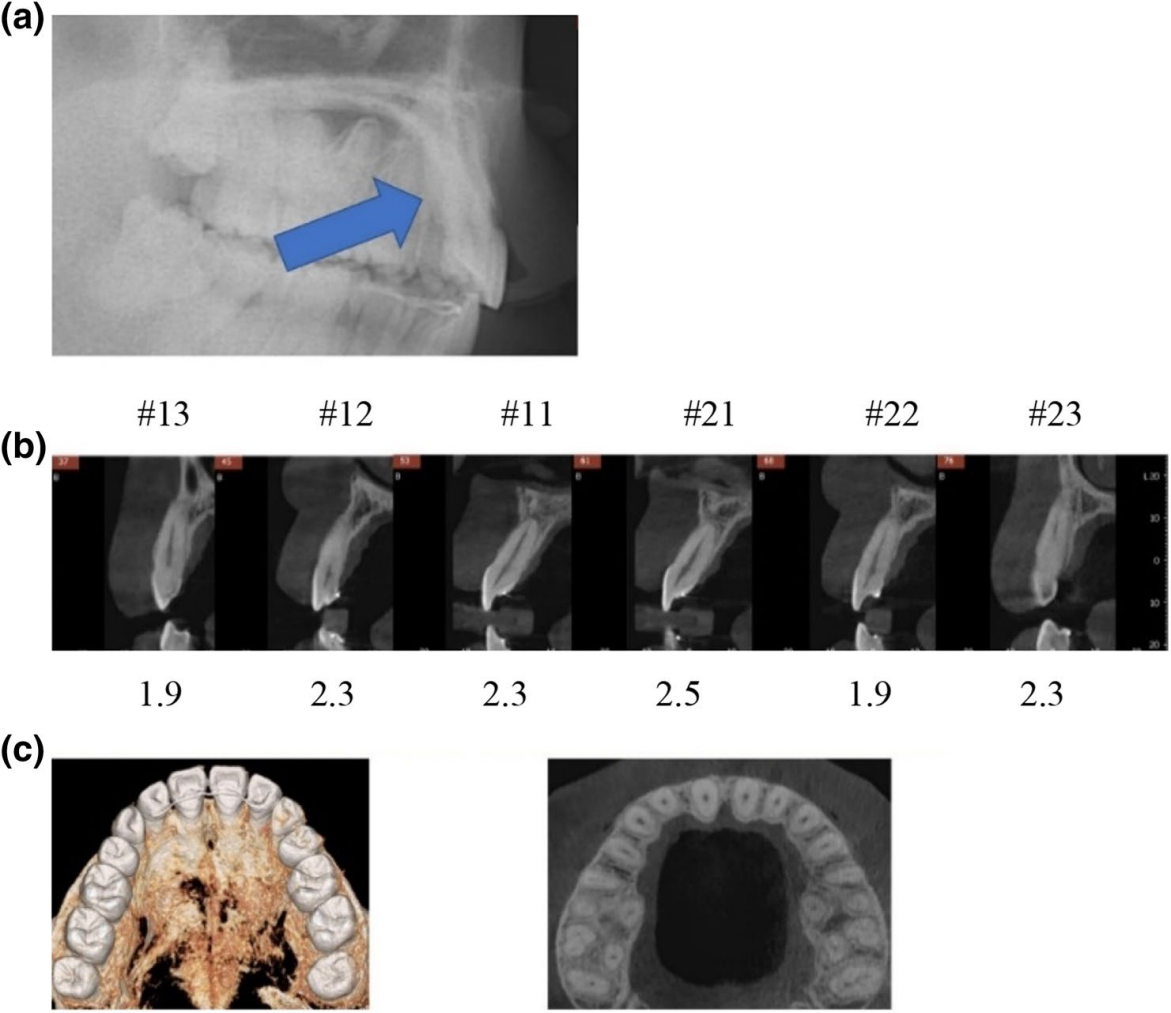
Another case longer than 5 years of retention; (**a**) debonding stage. Arrow indicates palatal bone loss; (**b**) numbers below the teeth indicate the distance from CEJ to the deepest point of alveolar crest; (**c**) the transverse view of the alveolar bone thickness after 5 years of retention.

Based on the CT detection of periodontal bone loss study^20^, a distance longer than three millimeters from the cementoenamel junction to the deepest point of alveolar bone indicated significant periodontal bone loss^20^. Examinations of 11 patients from the right maxillary canine to the left maxillary canine with a CBCT and software revealed that 26% (17 out of 66) of the teeth presented with vertical bone loss, and three out of eleven displayed more than two teeth involved. Figures 8 and 9 describes two cases with noticeable palatal bone loss at the debonding stage (8A and 9A). Figure 8 showed vertical bone loss remained except upper maxillary incisors, while normal alveolar bone level in incisors appears in Fig. 9.

## Discussion

Previous studies have shown that extraction treatment has a greater effect on root resorption and alveolar bone loss than non-extraction treatment^12^, and in extraction treatment, the inclination and position of the anterior teeth played an important role in the stability of the treatment and function. In recent years there are hypotheses that bone remodeling cannot follow exactly in synchronization with tooth movement in many orthodontic treatment modalities. Some authors explained that if the balance between resorption and apposition of the alveolar bone is not established during tooth movement, the tooth will move out of the alveolar housing, which is referred to as “through-the-bone”^5,22^. In adult extraction orthodontic treatment, it is generally accepted that alveolar bone loss and root resorption will result to some degree. However, many orthodontists state that excessive labial and lingual movement of incisors in the maxilla and mandible should be avoided due to the accompanying loss of supporting alveolar bone^11^. Skeletal anchorage in orthodontic treatment is clearly a useful treatment modality in proper indications, but excessive retraction and intrusion of teeth will cause overloading and result in loss of periodontal tissue and alveolar bone^5^. Previous studies have examined the shape and thickness of the labial side and palatal side of the alveolar bone around the maxillary and mandibular incisors^7^. The purpose of the present study was to evaluate the change in palatal alveolar bone thickness and the factors affecting the resorption of the palatal alveolar bone caused by tooth movement of the maxillary central incisors. A previous study indicated that while upper incisors were intruded and retracted simultaneously, various labial side response was described^23^. In the present study, the difference between labial and palatal response was evaluated. In comparing the angle between U1 and alveolar bone surface before and after treatment (Fig. 3, Table 2), the labial side showed more concurrent angular changes following the change in the inclination of teeth than the palatal side. The difference in thickness of the alveolar bone before and after treatment was significantly correlated with the amount of retraction of the maxillary central incisors. The thickness changed toward T1 (CEJ) indicating that more resorption is observed at the cervical area, but there was no significant ratio in the amount of retraction (Tables 3, 4). Similar results were found in the previous study^24^. The change in palatal alveolar bone thickness from T1 to T4 was correlated with the pre-treatment thickness, indicating that thin palatal alveolar bone at the CEJ before treatment requires careful monitoring during treatment.

**Figure 10:**
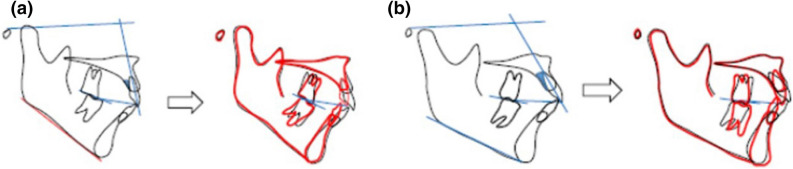
Cases expection greater palatal alveolar bone loss (**a**) and less bone loss (**b**).

Correlation analysis adopting the amount of palatal alveolar bone resorption (Fig. 6) as a dependent variable was performed to evaluate the effect of various angles at pre-treatment, post-treatment, and the change after the treatment. The analysis indicated that high angle class II with normal maxillary incisor inclination and the small palatal alveolar bone area was related to palatal alveolar bone resorption in premolar extraction treatment (Table 7, Fig. 10). Correlation analysis regarding post-treatment angles showed that lingually inclined maxillary incisors and a steeper occlusal plane of the maxilla and mandible (UOP, POP) were correlated with the amount of alveolar bone resorption (Table 7, Fig. 10). The correlation analysis regarding the change of angles during treatment showed that the steeper the occlusal plane after treatment, the greater the resorption of the palatal alveolar bone (Table 7, Fig. 10).

The age and the amount of retraction and intrusion of maxillary incisors were not significantly related with resorption of palatal alveolar bone. The occlusal plane of the maxilla and mandible (UOP, POP) before and after the treatment seemed to affect the resorption of palatal alveolar bone (Table 7, Fig. 10). This coincides with the previous findings that a steep occlusal plane in a Class II skeletal case will cause more retraction, and thus will affect palatal alveolar bone resorption if the occlusal plane remains steep or becomes even steeper after the extraction of premolars^22^. Therefore, for skeletal class II patients who are planned for mandibular plane counter-clockwise rotation, full arch intrusion rather than molars only intrusion is recommended for better preservation of palatal bone. For the follow-up study, 11 patients who had retention of longer than 5 years were asked to return to take CBCT. 26% of the patients (3/11) and teeth (17/66) demonstrated more than 3 mm distance between the CEJ and the deepest point of alveolar bone. The results showed that in ¼ of the cases, alveolar bone was not repaired, The bone level could possibly be affected by aging or other factors. Further studies will be required to evaluate the repairment In the present study, the average palatal bone loss amount was 2.5 mm, which is similar to other studies that evaluated palatal bone response after incisor retraction^5,9^. Since patients incorporated in this study were mostly gummy smile patients, an average of 1.9 mm of root apex intrusion was measured. Case reports presented marginal bone support enhancement after incisor intrusion in periodontally migrated teeth^25,26^. Therefore, it can be hypothesized that intrusion of incisors may compensate for vertical palatal bone loss during maxillary incisor intrusion and retraction. The results of this study did not support that hypothesis. Therefore, the palatal bone loss must be monitored carefully during the treatment.

There are a few limitations and areas of improvements in this study. Measurements were taken with lateral cephalometric radiographs, and further quantitative research based on 3D CT will greatly improve the higher resolution of the images and finer measurements. However, since not many orthodontists are using CBCT for initial diagnosis, lateral cephalometrix is also a practical tool to find factors related to alveolar bone remodeling. A higher radiation dose of CBCT is another concern^27^. Lastly, to compare alveolar bone contour change, the selection of cross-sectional sections can be subjective. Further research to investigate the factors related to palatal bone repair is necessary.

## Data Availability

The data supporting the study can be obtained directly from the authors.
